# Texture-based image feature analysis for the classification of oral squamous cell carcinoma using machine learning approach

**DOI:** 10.1186/s12903-025-07482-1

**Published:** 2025-12-27

**Authors:** Salma Bahaa, Omneya Ramadan, Zeinab Elsayed Darwish, Eslam EL-Fiky, Mai M. Saleh

**Affiliations:** 1 Oral Pathology, Faculty of Dentistry, Alexandria University, Alexandria, Champollion, Elazarita, 21563 Egypt; 2 Electrical Engineering, Faculty of Engineering, Alexandria University, Alexandria, 21544 Egypt

**Keywords:** Oral squamous cell carcinoma, Machine learning, Deep learning, Texture feature extraction

## Abstract

**Background:**

Artificial intelligence is being developed as a vital tool to improve the accuracy and efficiency of oral cancer diagnosis. Oral squamous cell carcinoma is often detected at advanced stages, leading to poor patient outcomes, and traditional diagnostic methods can be subjective and physically demanding for pathologists. By comparing different Artificial intelligence models, including machine learning and deep learning, this study aims to create a more standardized and rapid diagnostic aid that can support pathologists and increase patient survival rates.

**Methods:**

A total of 115 cases (49 OSCC, 66 normal) were captured at 100x and 400x magnifications. Twelve Gray Level Co-occurrence Matrix (GLCM) features were extracted. Machine learning models, including Fine Tree, Medium Tree, Linear Discriminant, Linear Support Vector Machine, K-Nearest Neighbors, and Neural Network (MLP) model were developed. Model performance was evaluated across four distinct classification tasks, assessing the contribution of histopathological images alone and in fusion with demographic/clinical data. Statistical validation involved 5-fold cross-validation on the training set and independent testing, reporting sensitivity, specificity, precision, F1 score, and overall accuracy.

**Results:**

Analysis of Gray Level Co-occurrence Matrix (GLCM) features revealed significant textural disparities between normal and OSCC. Specifically, OSCC images showed trends of finer texture (lower autocorrelation), higher local intensity variation, increased complexity (entropy), greater intensity disparities (dissimilarity), and reduced uniformity (lower energy, maximum probability, and homogeneity) compared to normal counterparts. Leveraging these distinct textural signatures, the developed machine learning models exhibited exceptional classification capabilities, frequently achieving 100% sensitivity, specificity, precision, F1 score, and overall accuracy across all tasks during both validation and testing of models. This high performance highlights the reliability of the proposed approach for differentiating OSCC.

**Conclusions:**

This study demonstrated that statistical image features effectively capture distinct textural differences between normal and OSCC tissues. The developed machine learning models showed outstanding classification performance, demonstrating their strong potential for accurate, automated OSCC diagnosis. This near-perfect accuracy could revolutionize diagnostic workflows, minimizing subjective interpretations and enhancing overall efficiency.

**Supplementary Information:**

The online version contains supplementary material available at 10.1186/s12903-025-07482-1.

## Background

 Artificial intelligence (AI) marks a pivotal moment in healthcare’s evolution, propelling advancements that are revolutionizing medical diagnostics [1]. The World Health Organization (WHO) reports that 389.846 new instances of oral and lip carcinoma were detected in 2022, ranking it as the sixteenth most frequent cancer and the fifteenth in mortality rate globally. In Egypt, there are 1578 new instances of oral and lip cancer ranking it as the eighteenth most frequent cancer and the eighteenth in mortality rate [2]. Oral squamous cell carcinoma (OSCC) is thought to account for about 90% all oral neoplasms [3, 4]. Overall mortality rate of OSCC has not significantly decreased since the 1980 s, despite the adoption of several novel treatment modalities over the past few decades [5]. This is because there has been relatively little effort put into screening and early detection, which explains the high diagnosis rate of diseases at their advanced stages [5]. Given that the predicted 5-year survival rate for OSCC shows a clear decline from 84% if discovered in its early stages (I and II) to roughly 39% if found in its advanced stages (III and IV), it is crucial to detect OSCC in early stages to improve patient outcomes [5, 6]. OSCC is typically detected at an advanced stage because of a lack of knowledge or access to adequate medical care. This is especially noticeable in nations with moderate or low incomes when access to healthcare is limited [7].

Histopathological examination of tissue samples using light microscope is regarded as the accepted technique for diagnosing OSCC [8, 9]. Pathologists frequently experience physical discomfort, such as dizziness and neck strain, due to the prolonged periods spent examining slides under a microscope. This manual analysis becomes particularly demanding and laborious given the increasing number of cancer patients requiring a large volume of tissue sections to be reviewed [10]. Moreover, an oral pathologist’s histological assessment of biopsies is subjected to subjective opinion because of variations in interpretation and inconsistent cutoff points in the morphological spectrum which result in inconsistent outcomes [11, 12].

Therefore, the established value of double-checking in histopathological diagnoses within clinical practice is needed. At the same time, alternative techniques are required in order to provide a diagnosis that is expected to be more efficient, quick, and standardized resulting in increase the survival rates of oral cancer patients [13].

Hence, employing AI as a form of verification process could enhance the precision of these diagnoses [13]. Contemporary research increasingly demonstrates the adoption of AI based image analysis methodologies for the assessment of routine histological specimens [14–25].

Artificial intelligence strives to enable computers to execute tasks that traditionally require human intellect, such as learning, problem-solving, and decision-making [26]. Specifically in this field, the creation of image analysis algorithms has driven the application of AI for more objective, timely, and precise cancer detection [27]. Generally, AI includes traditional machine learning and deep learning [28]. Traditional machine learning is divided into supervised learning, which uses labeled data, and unsupervised learning, which finds patterns in unlabeled data [29]. Deep learning, a specific type of machine learning also known as neural networks, uses multiple hidden layers to process large amounts of complex data and uncover intricate relationships [28].

There is an essential step of transforming data, from its initial unstructured form to organized datasets, to form valuable features for machine learning algorithms which is known as feature extraction. The objective of this process is to improve the precision of models by pinpointing and utilizing features that are insightful and significant, while decreasing noise and unnecessary repetition [30].

To achieve highly accurate classification, shape, color, and texture are indispensable characteristics. Gray Level Co-occurrence Matrix (GLCM) is a powerful method for feature extraction, effectively quantifying textural properties [31].

In this study, our objective was to develop different AI models including machine learning and deep learning to compare their precision and accuracy to help the oral pathologist in diagnosis of OSCC.

## Methods

A comparative diagnostic accuracy study was conducted in the Department of Oral Pathology, Faculty of Dentistry, Alexandria University, Egypt. This study was performed after gaining the approval of the Research Ethics Committee, Faculty of Dentistry, Alexandria University (IRB No. 001056- IORG 0008839).

### Sample size calculation

The sample size calculation was based on previous published research by Cheung et al., 2023 [32]. Based on a 95% confidence level and 80% study power to detect the level of accuracy; the required sample size was calculated to be 381, increased to 419 images to make up for processing problems. This number was for the histological images of OSCC and the same number for normal histological images [33].

The study was carried in two main stages. The first one was about collecting the histological images of patients accompanied by their demographic and clinical data. The second step was about creating, training and testing a computer-aided diagnostic model based on machine learning and deep learning for categorization and differentiation between samples of OSCC and normal tissue.

### Data collection and preparation

Serial sections of 4–5 μm retrieved from the tissue blocks of cases of OSCC and normal tissue stained with hematoxylin and eosin (H and E) stain. The tissue blocks were obtained from the archive of Oral Pathology Department, Faculty of Dentistry, Alexandria University, Egypt.

A total number of 115 cases, 49 cases of OSCC with different grades (low, intermediate and high) as a study group, and 66 cases with normal histology as a control group were included in the dataset. Among the 49 OSCC cases analyzed, the distribution is as follows: 37 cases (75.5%) are intermediate grade OSCC, 9 cases (18.4%) are high grade OSCC, and 3 cases (6.1%) are low grade OSCC. Histological images were captured by Olympus Microscope DP20 Camera attached to Olympus BX41-P Polarizing Light Microscope, California, USA using CellSens standard software.

**Fig. 1:**
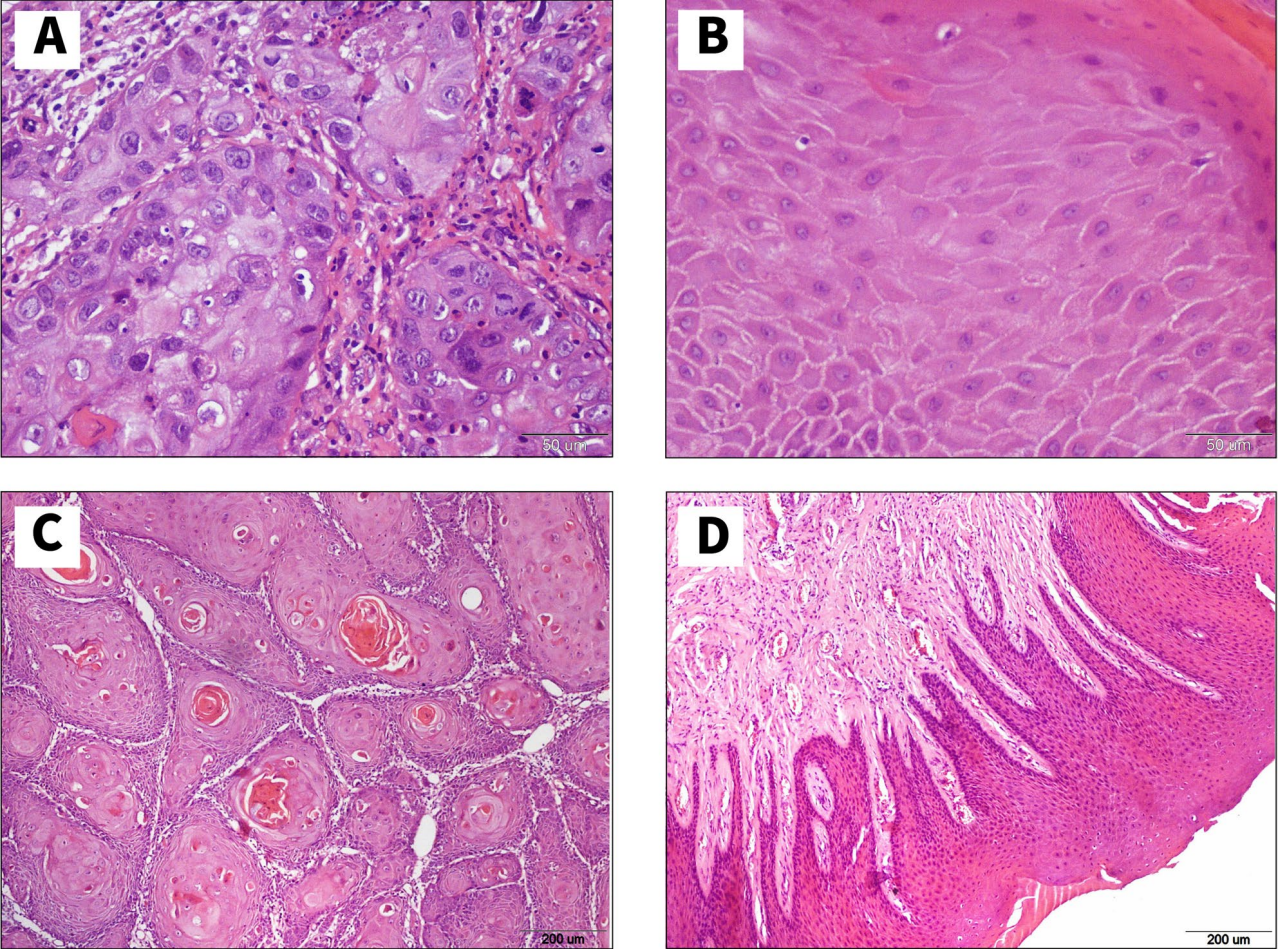
Histological images samples show (**A**) OSCC using objectives of 40 × (**B**) Normal epithelium using objectives of 40 × **(C)** OSCC using objectives of 10 × (**D**) Normal epithelium using objectives of 10 ×

Two sets of images were captured. The first set was captured with 40x objective lens with a total magnification of 400x to take a total number of 427 images of OSCC and 437 images of normal tissue Fig. 1A, B. The second set was captured with 10x objective lens with a total magnification of 100x to take a total number of 421 images of OSCC and 429 images of normal tissue Fig. 1C, D.

Sample size is defined by the number of diagnostic fields (images), not the total number of patients or original slides. Our initial work was image-based where image acquisition was designed to capture different diagnostic fields, confirmed by three independent pathologists and tracked by serial hospital numbers for every patient.

In the first set, we focused to detect the malignant criteria of squamous epithelial cells which are hyperchromatism, pleomorphism, altered nuclear cytoplasmic ratio, prominent nucleoli, normal and abnormal mitosis. In the second set, we focused to detect keratin pearls, epithelial pearls and cell nests and some of malignant criteria which could be seen at this magnification [34].

All images were saved as JPEG format in the size of 1,600 × 1,200 pixels [35]. To underscore the scope of this investigation, clinical and demographic data (age, gender, and the anatomical site of the lesion) were collected for each OSCC and control case [36, 37] as detailed in Table 1. Then, the images were standardized into 1100 × 1200 pixels. Then, white mask normalization was performed using a binary mask. Finally, the color normalization is performed on the RGB signal levels before merging to the image again. This was a necessary step to ensure computational efficiency and preserve consistency throughout the collection [35].

**Table 1:** Distribution of OSCC and normal samples by gender, age group, and anatomical site on image basis

Category	OSCC	Normal	Statistical Test	*P*-Value
Gender			**Chi-squared**	0.00022
Male	221	274		
Female	203	170		
Age Group			**Chi-squared**	< 0.00001
Group 0 (*<* 40 years)	4	170		
Group 1 (40–60 years)	225	270		
Group 2 (*>* 60 years)	195	4		
Anatomical Site			**Chi-squared**	< 0.00001
A. Buccal mucosa/Posterior buccal mucosa	25	30		
B. Mandibular alveolar ridge	83	94		
C. Lateral surface of tongue	173	38		
D. Ventral surface of tongue	40	0		
E. Upper lip	12	0		
F. Left maxilla/Anterior maxilla/Maxillary alveolar ridge	46	0		
G. Hard palate/Palatal surface of left maxilla	36	0		
H. Retromolar area	61	0		
I. Zygoma/Condyle	23	0		
J. Dorsal surface of tongue	0	17		
K. Anterior gingiva	0	265		

In Table 1, the analysis confirms that Gender (*p* = 0.00022), Age Group (*p* < 0.00001), and Anatomical Site (*p* < 0.00001) are all highly statistically significant factors differentiating the two groups. As these clinical variables show a strong association with the diagnosis, they were logically selected and entered as the full set of predictors.

### Image feature extraction

The images were processed to extract twelve texture features from the Gray- Level Co-occurrence Matrix (GLCM). It generates a matrix that captures the distribution of various gray levels within a designated region of interest. By analyzing how often different gray levels appear together (their co-occurrence), GLCM extracts distinct texture features. Within this region, both smooth and rough textures can be present. A smooth texture indicates that adjacent pixels have similar intensity values, while a rough texture signifies a notable difference between neighboring pixel values. Ultimately, GLCM utilizes spatial information to compute a variety of statistical metrics that characterize these textures [31]. This was performed by an outsourced software development built based on the image processing toolbox of MATLAB^®^ (The Math- Works Inc., Natick, MA, USA) 2024a platform. Images features were Autocorrelation, Contrast, Difference variance, Cluster Shade, Dissimilarity, Energy, Entropy, Sum entropy, Difference entropy, Homogeneity, Maximum Probability, and Information measure of correlation [32].

### Model development, validation and testing then integration of clinical and demographic data

The images were sent to the classification learner application in Matlab 2024a for model development after being processed for extracting the fore-mentioned GLCM image features.

Various algorithms were used to develop the model, including the Fine Tree (FT), Linear Discriminant (LD), K-Nearest Neighbors (KNN), Medium Tree (MT), Neural Network (a multilayer perceptron/MLP), and Linear Support Vector Machine (SVM). For Neural Network (MLP) model, the classifier utilized a fully connected feed-forward architecture designed to process 27 input features (13 GLCM features plus 14 clinical data points). The network structure consisted of two fully connected layers containing 64 and 2 neurons, respectively, employing a Sigmoid activation function between layers. Training was conducted using MATLAB’s Classification Learner, where Bayesian Optimization was used for hyperparameter tuning over 30 optimization iterations, searching across 1–3 layers, various activations (ReLU, Tanh, Sigmoid, None), regularization strengths (1.43 × 10⁻⁸ – 142.65), and layer sizes (1–300 neurons). The final chosen model incorporated L2 weight decay regularization with a strength of λ = 1.1509 × 10⁻⁷ to reduce overfitting. The model was trained for up to 1000 iterations using a training setup that implemented an early stopping configuration after 6 validation failures, and no data augmentation was applied.

Our pipeline involves a distinct feature extraction step where GLCM features are computed from the histological images, resulting in a vector/tabulated data input. MLPs are inherently well-suited for processing vectorized or tabulated data, making them appropriate for our design where feature vectors (GLCM features + clinical data) are the primary input, not the raw pixel data. Moreover, the use of an MLP with a Sigmoid activation function allows the network to effectively learn complex non-linear interactions and combinations between the extracted GLCM features and the clinical data, which is crucial for robust classification.

Every model underwent training, validation and testing. The AI model’s performance was evaluated using a two-step process.

To integrate image-derived features with demographic and clinical variables, multi-modal fusion techniques were employed [38].

This study was comprised of four separate classification tasks; three were conducted on the first dataset, and the fourth on the second dataset.

### Computational experiments

#### Task I- first set

 Classification using only histopathological images to distinguish between normal and OSCC samples with fusion of demographic and clinical data

#### Task II- first set

 Classification using only histopathological images to distinguish between normal and OSCC samples, as no demographic or clinical data were included.

#### Task III- first set

 Classification using only demographic and clinical data.

#### Task IV- second set

 Classification using only histopathological images to distinguish between normal and OSCC samples, as no demographic or clinical data were included.

Our work -for the all tasks- is initially image-based, then we have re-run the cross-validation with the patient-wise split to ensure our results are not inflated by data leakage and near-duplicate views. Furthermore, to demonstrate the consistency and robustness of our model within the cohort, the final reported results are supported by four additional, independent full cross-validation runs, each utilizing a new and distinct patient-level test set.

### Confounding mitigation and robustness testing

The retrospective nature of our data collection led to an imbalance in certain clinical variables, notably the anatomical site (e.g., Anterior Gingiva), which raised concerns that these variables could inadvertently encode the label. To mitigate the risk of patient-specific bias and confounding, we implemented two key procedural changes:

1. Patient-Level Data Splitting: All cross-validation and final test sets were strictly separated at the patient level. This ensured that images from the same patient were never split across both the training and testing partitions.
2. Clinical-Only Model Robustness Check: To validate the discriminatory power of clinical features independent of image count per patient, we performed an additional test on the Clinical-Only model using a highly constrained dataset consisting of only one randomly selected image per patient.

### Clinical feature ablation protocol

To rigorously quantify the contribution of individual clinical features, particularly the most influential ones, we conducted a dedicated feature importance and ablation study on the Clinical-Only model.

First, an initial assessment of feature relevance was performed using the Chi-Square (χ2) algorithm to rank clinical features by their importance in predicting the outcome.

**Fig. 2:**
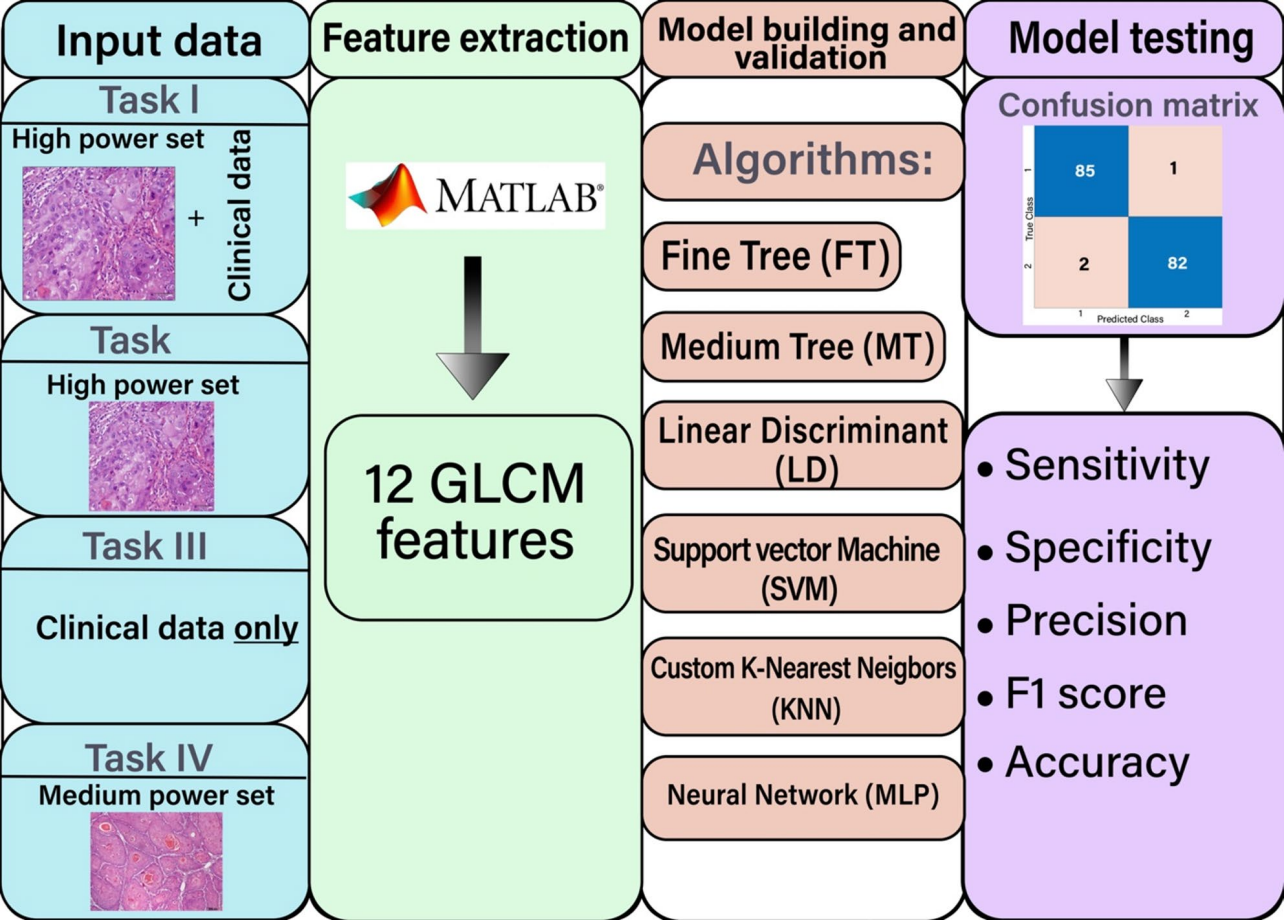
Study workflow

Figure 2 is an illustration diagram for the study workflow.

### Statistical analysis

To make the system less prone to overfitting and better at generalizing; the data was split, using 80% for training the model and keeping the remaining 20% aside as a completely separate test set. Then, to further assess the model’s learning on the training data, 5-fold cross-validation was employed. This process generated five different trained versions of the model. Finally, the performance of each of these five models was measured on the initial, untouched test set and reported the average performance along with the variability (standard deviation) across these five evaluations [39]. McNemar test was used to assess the difference between the evaluation of each model and the oral pathologist’s diagnosis. The performance of all computational tasks was statistically evaluated using sensitivity, specificity, precision, F1 score and overall accuracy. Metrics were presented as the mean and standard deviation of 5 runs for each pre-trained model used and each fusion method.

## Results

### Gray Level Co-occurrence Matrix (GLCM) image texture features

Texture feature is how an image looks uniform, showing the surface details of an object, whether they change slowly or quickly. Texture features describe these surface qualities, like how rough or dense an image looks. It’s a general feature, not affected by color or brightness, and it’s popular for image analysis because it’s resistant to rotation and noise. GLCM is a highly effective and commonly used method for extracting texture features. Essentially, GLCM works by showing how often pairs of pixels with specific brightness levels appear together at a certain distance and angle from each other [10].

**Fig. 3:**
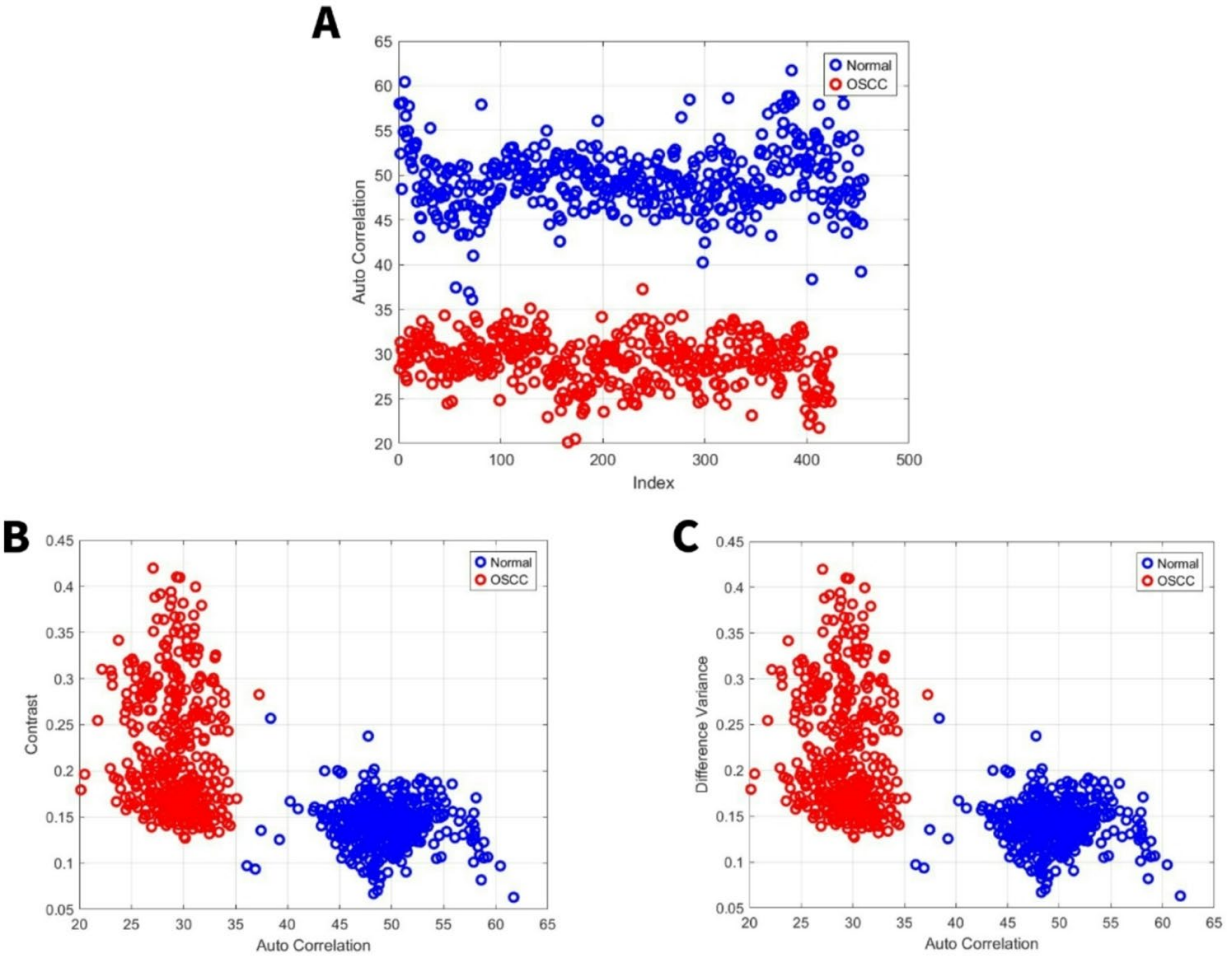
GLCM image features for high power set. **A** Autocorrelation. **B** Contrast vs. autocorrelation. **C** Difference Variance vs. autocorrelation

**Fig. 4:**
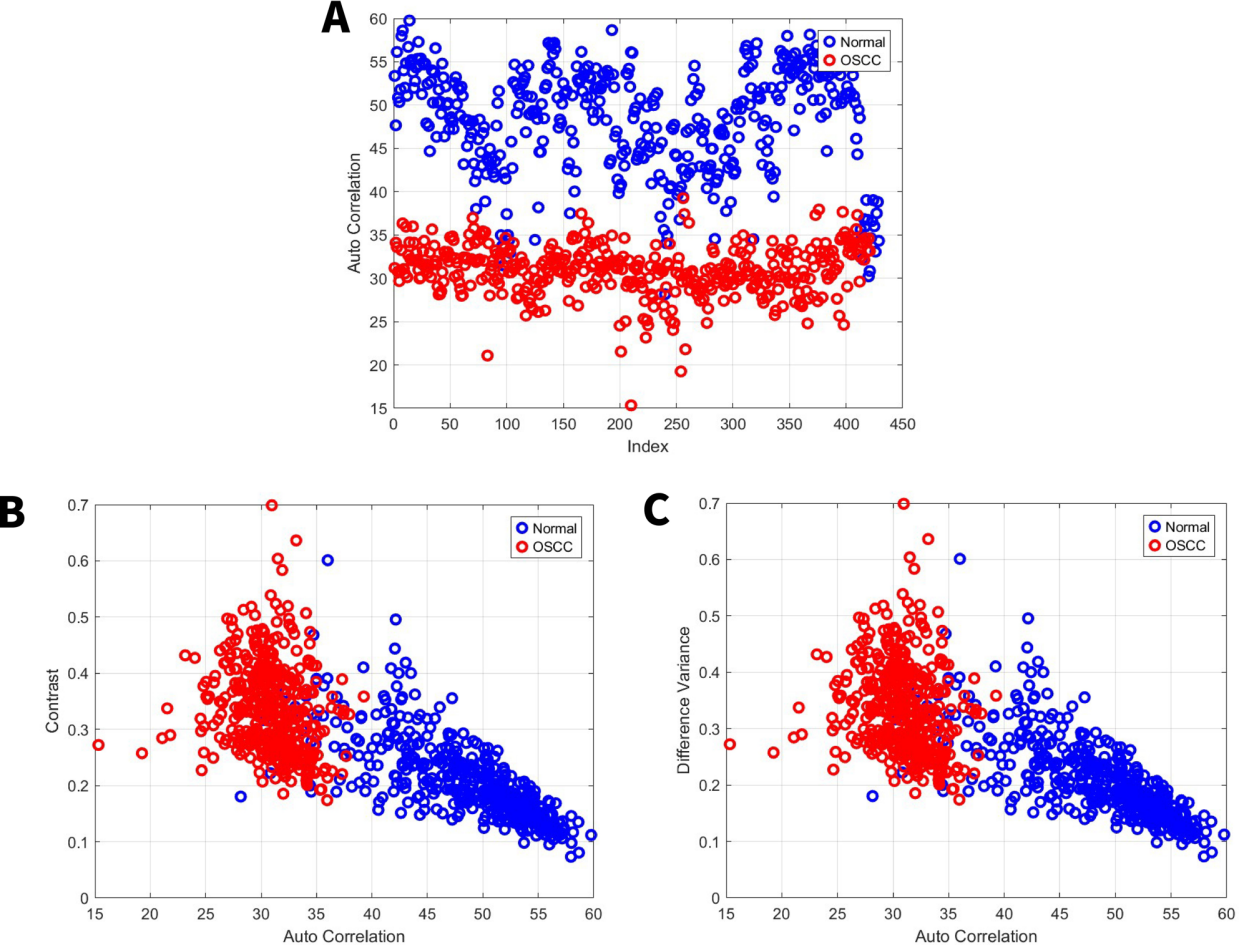
GLCM image features for medium power set. **A** Autocorrelation. **B** Contrast vs. autocorrelation. **C** Difference Variance vs. autocorrelation

 Autocorrelation quantifies texture magnitude, indicating fineness (smaller values) or coarseness. A smaller autocorrelation range suggests finer image details [40]. For the first set, the normal group exhibited an autocorrelation range from 36 to 62, while the OSCC group’s range was 20 to 37 Fig. 3A. For the second set, the normal group exhibited an autocorrelation range from 27 to 60, while the OSCC group’s range was 15 to 40 Fig. 4A. To standardize the presentation of image features using autocorrelation. All other image features were shown in comparison to autocorrelation.

### Image features related to local intensity variation

Contrast and difference variance are image features associated with local intensity. Specifically, contrast within the GLCM reflects the intensity difference between neighboring pixels, with high contrast indicating rapid changes in intensity [40, 41]. For the first set, the normal group exhibited a local intensity range from 0.06 to 0.26, while the OSCC group’s range was 0.13 to 0.43 Fig. 3B, C. For the second set, the normal group exhibited a local intensity range from 0.07 to 0.6, while the OSCC group’s range was 0.17 to 0.7 Fig. 4B, C

### Image features related to entropy

**Fig. 5:**
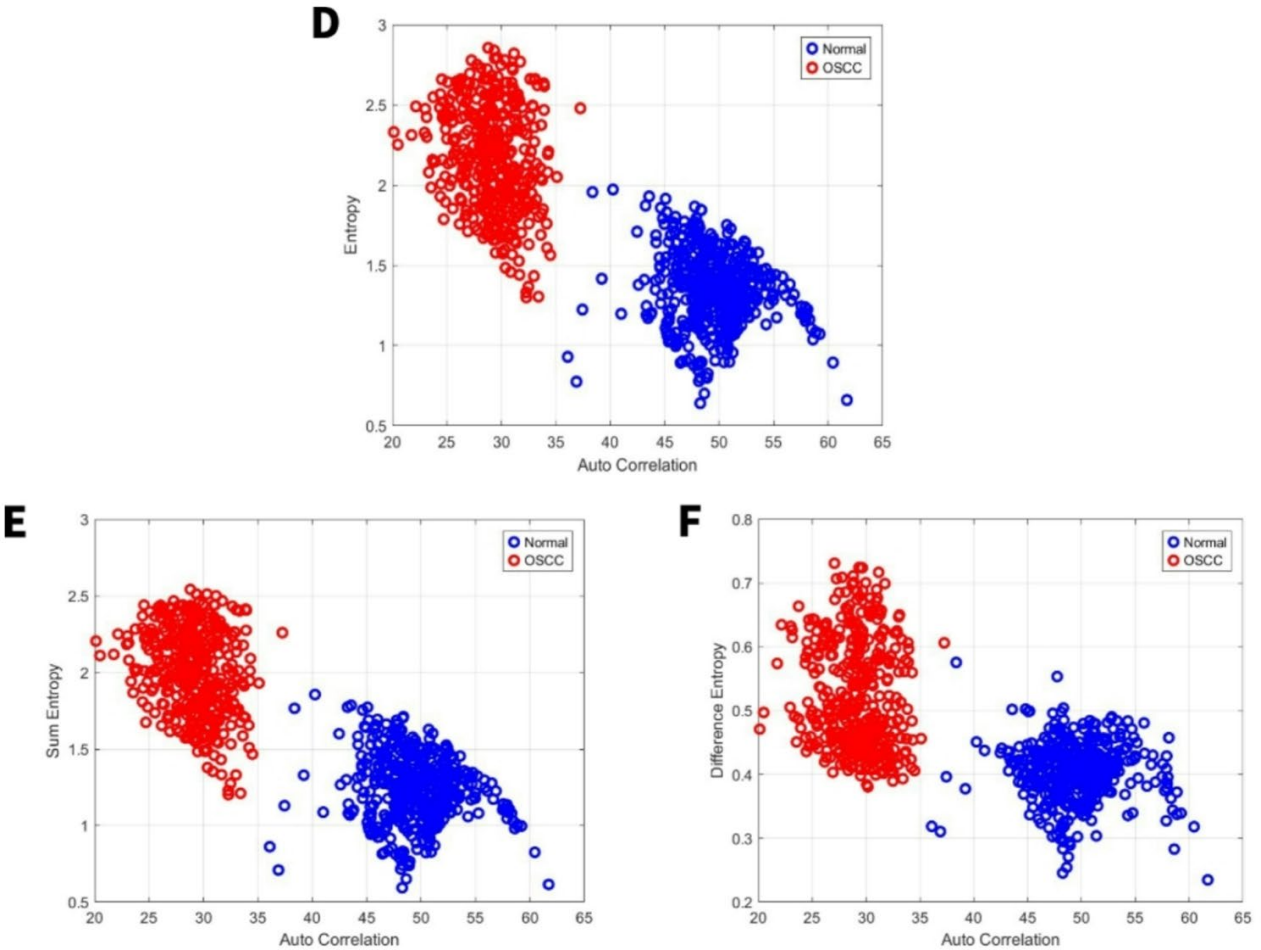
GLCM image features for high power set. **D** Entropy vs. autocorrelation. **E** Sum Entropy vs. autocorrelation. **F** Difference Entropy vs. autocorrelation

**Fig. 6:**
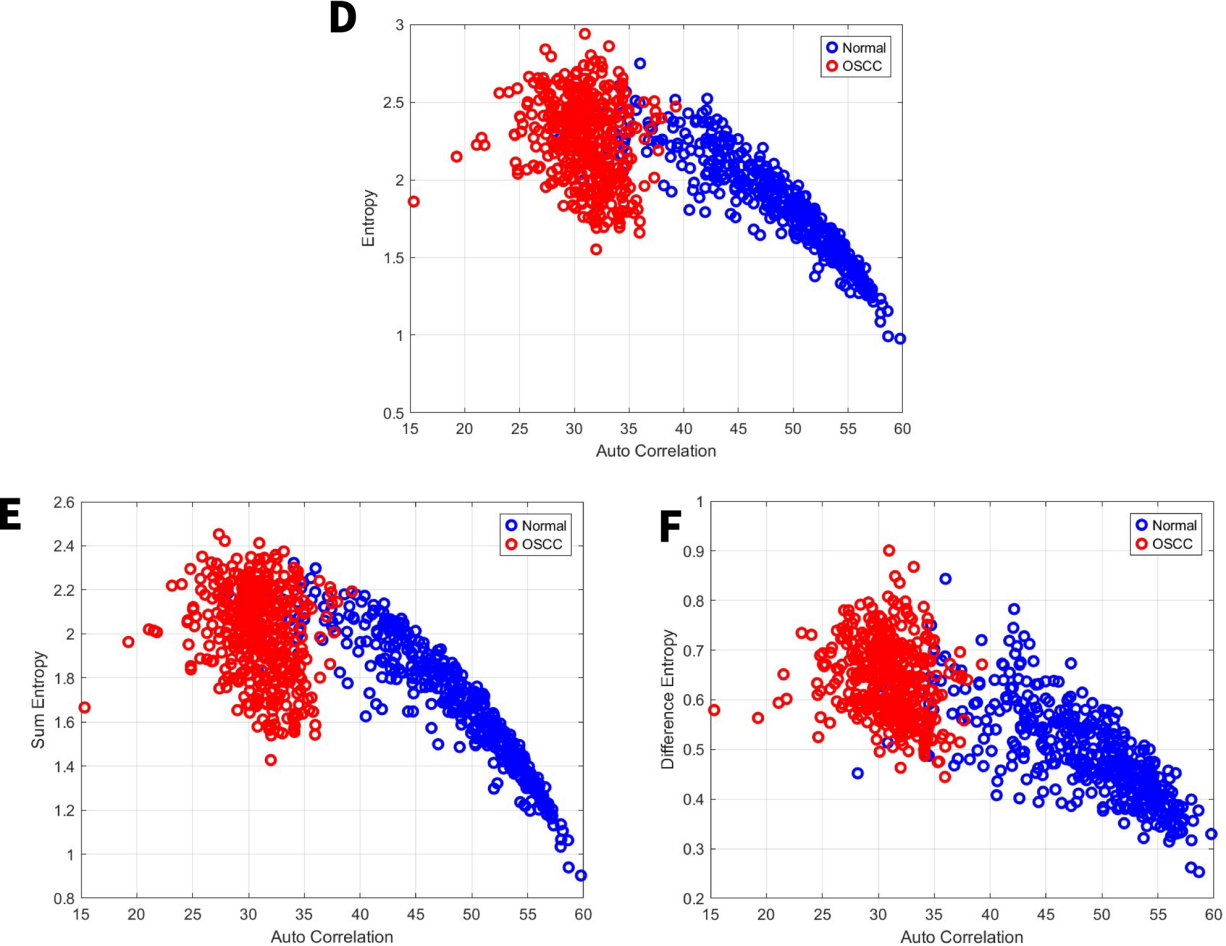
GLCM image features for medium power set. **D** Entropy vs. autocorrelation. **E** Sum Entropy vs. autocorrelation. **F** Difference Entropy vs. autocorrelation

Entropy quantifies image randomness; higher entropy signifies greater complexity and randomness [40, 41]. For the first set, our study showed that the entropy, sum entropy, and difference entropy range from 0.6 to 2, 0.6 to 1.8, and 0.22 to 0.58 for the normal group and range from 1.3 to 2.8, 1.2 to 2.6, and 0.38 to 0.74 for the OSCC group, respectively Fig. 5D, E,F. For the second set, our study showed that the entropy, sum entropy, and difference entropy range from 0.95 to 2,8, 0.9 to 2.35, and 0.25 to 0.85 for the normal group and range from 1.5 to 3, 1.4 to 2.5, and 0.44 to 0.9 for the OSCC group, respectively Fig. 6D, E,F.

### Image feature related to dissimilarity

**Fig. 7:**
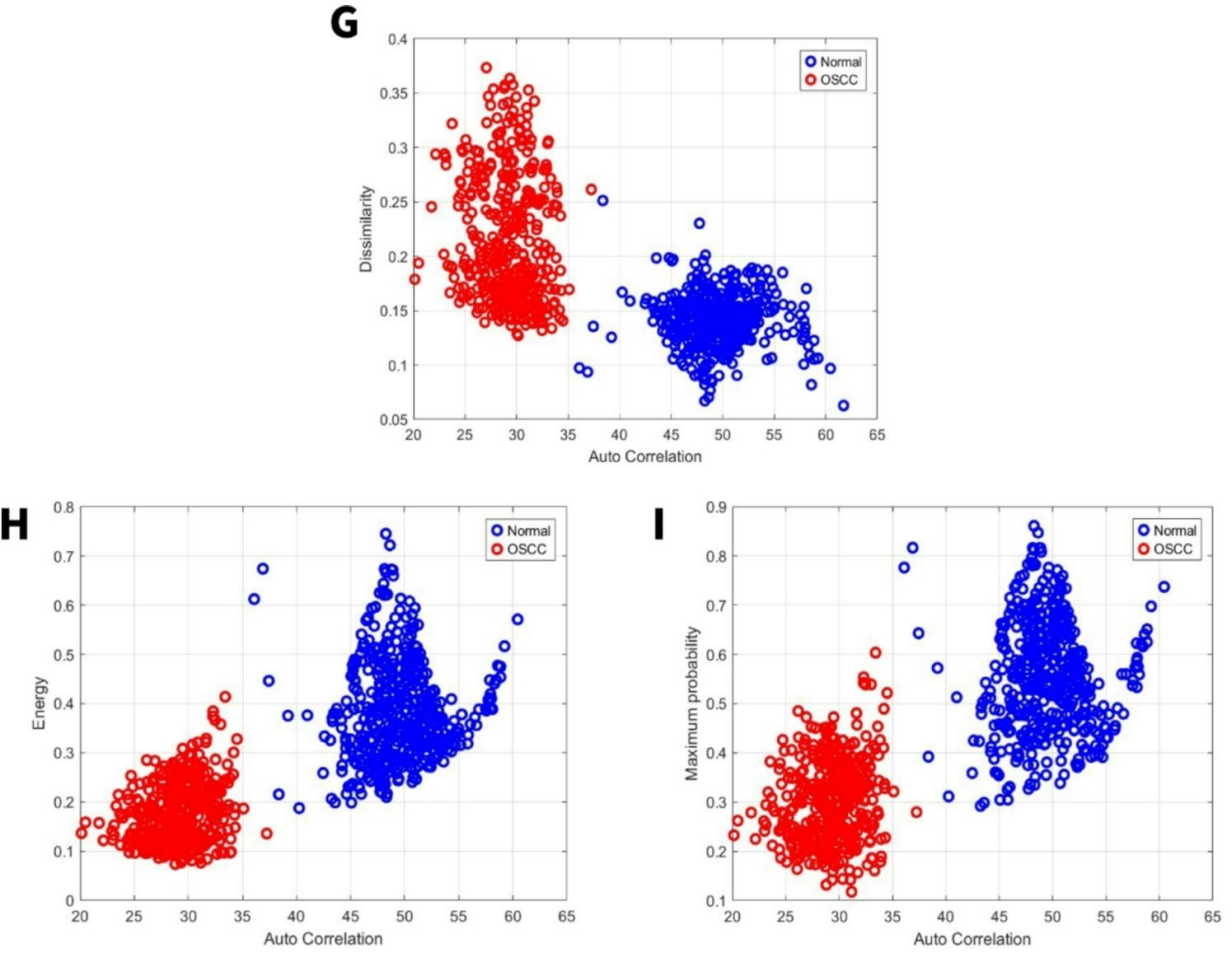
GLCM image features for high power set. **G** Dissimilarity vs. autocorrelation. **H** Energy vs. autocorrelation. **I** Maximum probability vs. autocorrelation

**Fig. 8:**
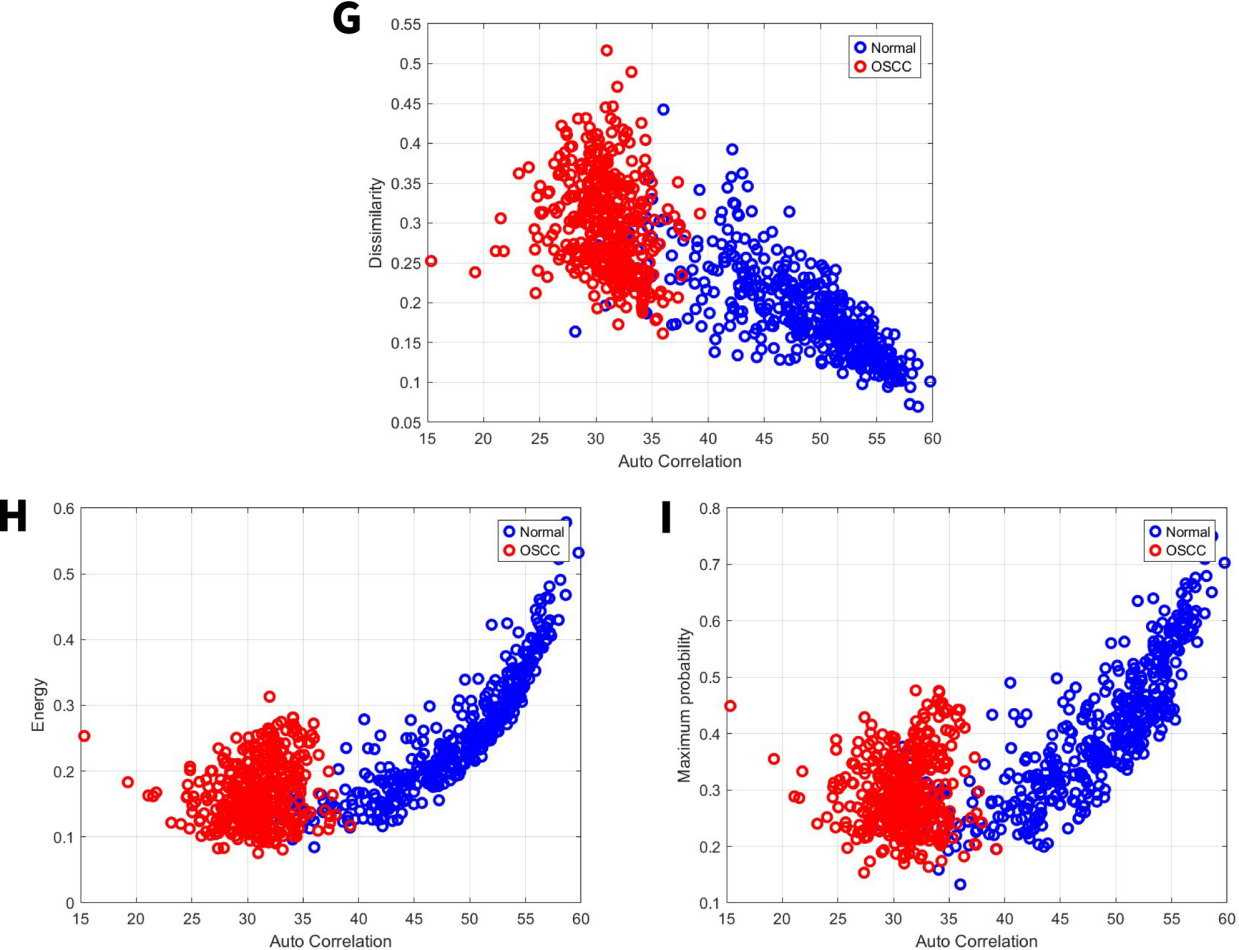
GLCM image features for medium power set. **G** Dissimilarity vs. autocorrelation. **H** Energy vs. autocorrelation. **I** Maximum probability vs. autocorrelation

Dissimilarity compares how often pixel pairs with different intensity values appear relative to pairs with the same intensity [40]. For the first set, the normal group exhibited a dissimilarity range from 0.06 to 0.25, while the OSCC group’s range was 0.13 to 0.38 Fig. 7G. For the second set, the normal group exhibited a dissimilarity range from 0.06 to 0.45, while the OSCC group’s range was 0.16 to 0.53 Fig. 8G. When there is more dissimilarity, it means the average brightness levels in different parts of the image are more distinct.

### Image feature energy and maximum probability

By applying local masks within a defined window, energy measures the degree of intensity change, enabling the identification of regions with uniform intensity (level), boundaries (edges), small areas (spots), and wave-like patterns (ripples) [32]. For the first set, the normal group exhibited an energy range from 0.19 to 0.75, while the OSCC group’s range was 0.08 to 0.43 Fig. 7H. For the second set, the normal group exhibited an energy range from 0.08 to 0.58, while the OSCC group’s range was 0.08 to 0.32 Fig. 8H. For the first set, the normal group exhibited a maximum probability range from 0.3 to 0.88, while the OSCC group’s range was 0.11 to 0.6 Fig. 7I. For the second set, the normal group exhibited a maximum probability range from 0.12 to 0.75, while the OSCC group’s range was 0.15 to 0.49 Fig. 8I. Higher energy levels reflect a greater tendency for neighboring intensity values to co-occur at higher frequencies. Similarly, maximum probability assessed the prevalence of the most frequent adjacent intensity value pairing.

### Image features related to homogeneity

**Fig. 9:**
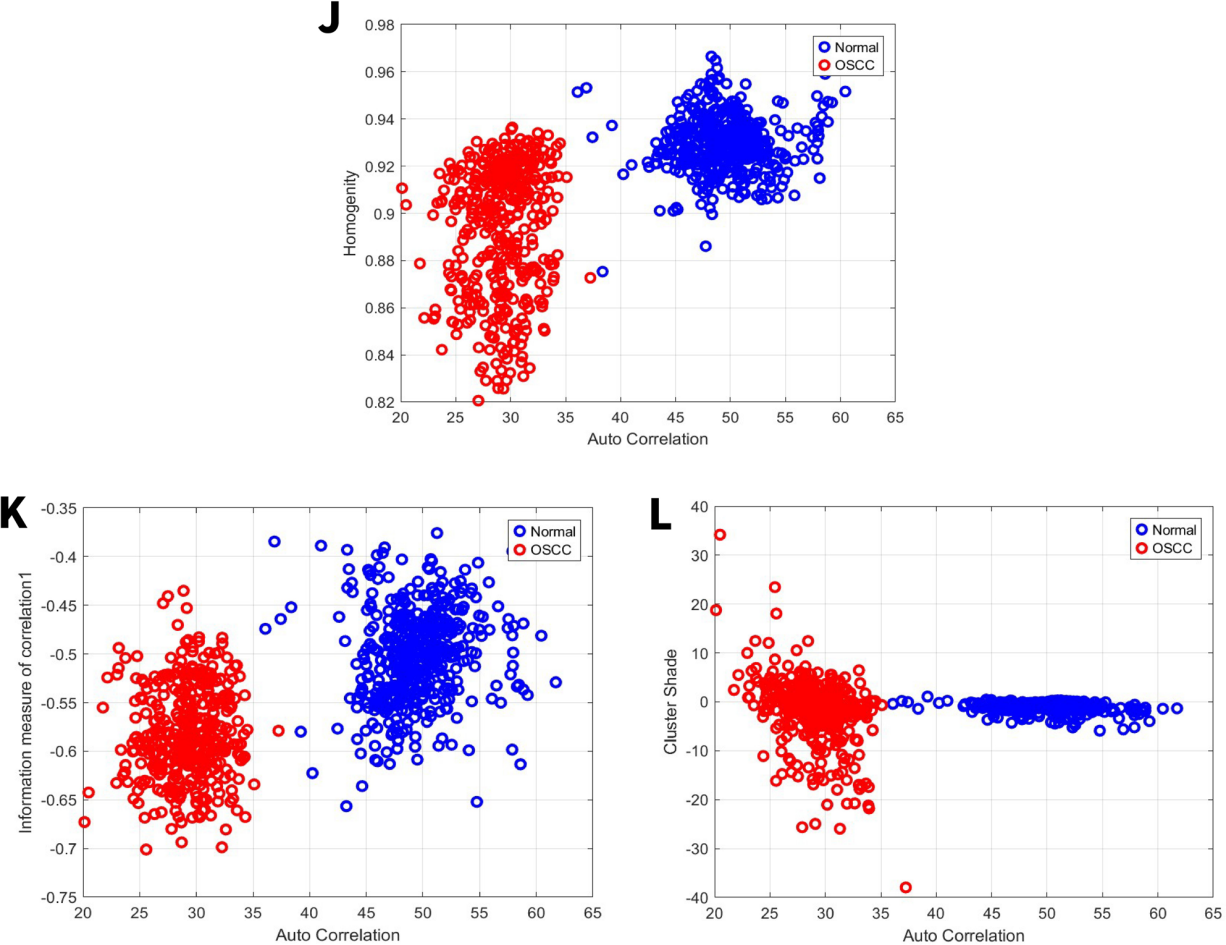
GLCM image features for high power set. **J** Homogeneity vs. autocorrelation. **K** Information measure of correlation 1 vs. autocorrelation. **L** Cluster shade vs. autocorrelation

**Fig. 10:**
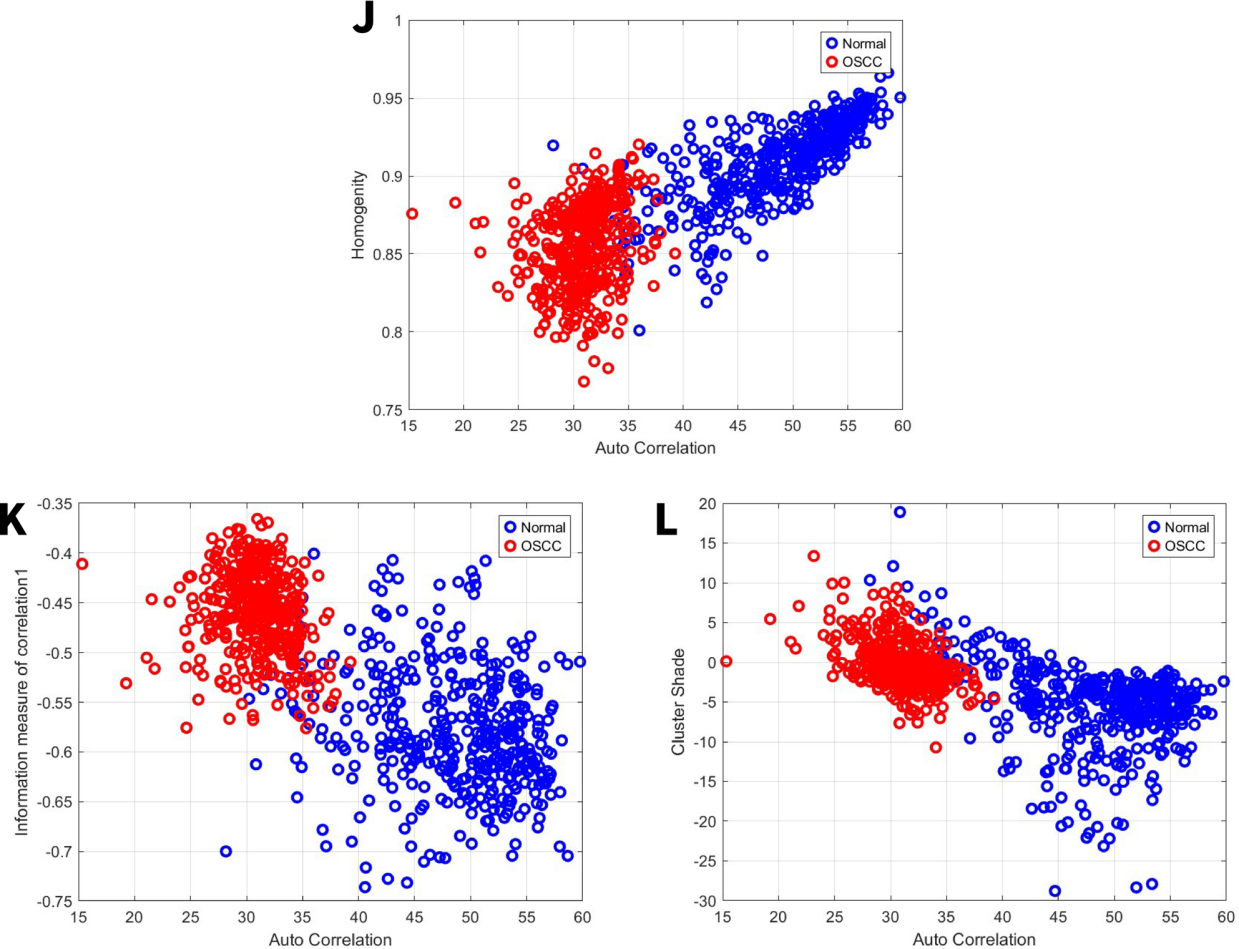
GLCM image features for medium power set. **J** Homogeneity vs. autocorrelation. **K** Information measure of correlation 1 vs. autocorrelation. **L** Cluster shade vs. autocorrelation

Homogeneity assesses the similarity or consistency of intensity values between adjacent pixels [42]. For the first set, the normal group exhibited a homogeneity ranges from 0.868 to 0.965, while the OSCC group’s range was 0.82 to 0.94 Fig. 9J. For the second set, the normal group exhibited a homogeneity range from 0.8 to 0.954, while the OSCC group’s range was 0.77 to 0.93 Fig. 10J. As the value increases, the image intensity becomes more uniform.

### Image features related to information measure of correlation

Information measure of correlation measures how dependent the gray levels of pixel pairs are in an image, using concepts from information theory (entropy). It tells us if the value of one pixel can predict the value of another. Information measure of correlation 1 measures linear dependence between pixel pairs. High information measure of Correlation 1 means that pixels are strongly correlated (predictable). Low information measure of Correlation 1 means that pixels are independent (random) [43]. For the first set, the normal group exhibited information measure of correlation ranges from − 0.37 to −0.66, while the OSCC group’s range was − 0.43 to −0.7 Fig. 9K. For the second set, the normal group exhibited information measure of correlation ranges from − 0.4 to −0.74, while the OSCC group’s range was − 0.36 to −0.57 Fig. 10K.

### Image features related to cluster shade

Cluster shade, a metric for the matrix’s skewness, is understood to assess the perceived uniformity of textural patterns [44]. For the first set, the normal group exhibited a cluster shade ranges from − 5 to 1, while the OSCC group’s range was − 39 to 35 Fig. 9L. For the second set, the normal group exhibited a cluster shade range of −29 to 20, while the OSCC group’s range was − 11 to 14 Fig. 10L. 

### Results of task

#### Models building and validation for all tasks

The results of Task I presented in Table 2 showed the highest sensitivity, specificity, precision, F1 score and Overall accuracy was 100% obtained by 4 models, which are medium tree, SVM, KNN and Neural Network (MLP).

**Table 2:** Sensitivity, Specificity, Precision, F1 score and accuracy in model validation for high power set with clinical data

Algorithm	Sensitivity	Specificity	Precision	F1 Score	Accuracy
Fine tree	99.72%	99.41%	99.45%	99.59%	99.57%
Medium tree	100.00%	100.00%	100.00%	100.00%	100.00%
Discriminant	99.72%	99.7%	99.72%	99.72%	99.57%
SVM	100.00%	100.00%	100.00%	100.00%	100.00%
KNN	100.00%	100.00%	100.00%	100.00%	100.00%
Neural Network (MLP)	100.00%	100.00%	100.00%	100.00%	100.00%

The results of Task II presented in Table 3 showed that the highest sensitivity, specificity, precision, F1 score and overall accuracy was 100% obtained by two models which are KNN and Neural Network (MLP).

**Table 3:** Sensitivity, Specificity, Precision, F1 score and accuracy in model validation for high power set without clinical data

Al Algorithm	Sensitivity	Specificity	Precision	F1 Score	Accuracy
Fine tree	99.72%	99.71%	99.72%	99.72%	99.72%
Medium tree	99.72%	99.71%	99.72%	99.72%	99.72%
Discriminant	99.86%	99.71%	99.86%	99.86%	99.86%
SVM	99.72%	99.42%	99.72%	99.72%	99.72%
KNN	100.00%	100.00%	100.00%	100.00%	100.00%
Neural Network (MLP)	100.00%	100.00%	100.00%	100.00%	100.00%

The results of Task III presented in Table 4 showed the same sensitivity, specificity, precision, F1 score and Overall accuracy was 100% obtained by Fine tree, Medium tree, KNN and Neural Network (MLP).

**Table 4:** Sensitivity, Specificity, Precision, F1 score and accuracy in model validation for high power set for clinical data only

Algorithm	Sensitivity	Specificity	Precision	F1 Score	Accuracy
Fine tree	100.00%	100.00%	100.00%	100.00%	100.00%
Medium tree	100.00%	100.00%	100.00%	100.00%	100.00%
Discriminant	88.38%	80.96%	89.55%	8*7.96*%	88.02%
SVM	99.26%	100.00%	99.32%	99.29%	99.29%
KNN	100.00%	100.00%	100.00%	100.00%	100.00%
Neural Network (MLP)	100.00%	100.00%	100.00%	100.00%	100.00%

The results of Task IV presented in Table 5 showed that the highest sensitivity was 100% obtained by two models which were Discriminant and Neural Network (MLP), while the highest specificity, precision, F1 score and overall accuracy was 100% obtained by discriminant model.

**Table 5:** Sensitivity, Specificity, Precision, F1 score and accuracy in model validation for medium power

Algorithm	Sensitivity	Specificity	Precision	F1 Score	Accuracy
Fine tree	96.51%	96.43%	98.81%	97.65%	96.47%
Medium tree	96.51%	96.43%	96.51%	96.51%	96.47%
Discriminant	100.00%	100.00%	100.00%	100.00%	100.00%
SVM	97.67%	97.62%	98.82%	98.25%	97.65%
KNN	97.59%	94.25%	94.19%	95.86%	95.88%
Neural Network (MLP)	100.00%	98.82%	98.84%	99.42%	99.41%

#### Models testing for all tasks

The results of Task I presented in Table 6 showed the same sensitivity, specificity, precision, F1 score and Overall accuracy was 100% obtained by the 6 models.

**Table 6:** Sensitivity, Specificity, Precision, F1 score and accuracy in model test for high power set with clinical data

Algorithm	Sensitivity (95% CI)	Specificity (95% CI)	Precision (95% CI)	F1 score	Accuracy (95% CI)
Fine tree	100.00 (96.03, 100.00)	100.00 (95.70, 100.00)	100.00 (96.03, 100.00)	100.00	100.00 (97.91, 100.00)
Medium tree	100.00 (96.03, 100.00)	100.00 (95.70, 100.00)	100.00 (96.03, 100.00)	100.00	100.00 (97.91, 100.00)
Discriminant	100.00 (96.03, 100.00)	100.00 (95.70, 100.00)	100.00 (96.03, 100.00)	100.00	100.00 (97.91, 100.00)
SVM	100.00 (96.03, 100.00)	100.00 (95.70, 100.00)	100.00 (96.03, 100.00)	100.00	100.00 (97.91, 100.00)
KNN	100.00 (96.03, 100.00)	100.00 (95.70, 100.00)	100.00 (96.03, 100.00)	100.00	100.00 (97.91, 100.00)
Neural Network (MLP)	100.00 (96.03, 100.00)	100.00 (95.70, 100.00)	100.00 (96.03, 100.00)	100.00	100.00 (97.91, 100.00)

*CI* Confidence interval

The results of Task II presented in Table 7 showed the same sensitivity, specificity, precision, F1 score and Overall accuracy was 100% obtained by the 6 models.

**Table 7:** Sensitivity, specificity, precision, F1 score, and accuracy in model test for high power set without clinical data

Algorithm	Sensitivity (95% CI)	Specificity (95% CI)	Precision (95% CI)	F1 score	Accuracy (95% CI)
Fine tree	100.00 (96.03, 100.00)	100.00 (95.70, 100.00)	100.00 (96.03, 100.00)	100.00	100.00 (97.91, 100.00)
Medium tree	100.00 (96.03, 100.00)	100.00 (95.70, 100.00)	100.00 (96.03, 100.00)	100.00	100.00 (97.91, 100.00)
Discriminant	100.00 (96.03, 100.00)	100.00 (95.70, 100.00)	100.00 (96.03, 100.00)	100.00	100.00 (97.91, 100.00)
SVM	100.00 (96.03, 100.00)	100.00 (95.70, 100.00)	100.00 (96.03, 100.00)	100.00	100.00 (97.91, 100.00)
KNN	100.00 (96.03, 100.00)	100.00 (95.70, 100.00)	100.00 (96.03, 100.00)	100.00	100.00 (97.91, 100.00)
Neural Network (MLP)	100.00 (96.03, 100.00)	100.00 (95.70, 100.00)	100.00 (96.03, 100.00)	100.00	100.00 (97.91, 100.00)

*CI* Confidence interval

The results of Task III presented in Table 8 showed the highest sensitivity, specificity, precision, F1 score and overall accuracy was 97.85%, 95.56%, 97.78%, 97.77%, 97.77% respectively obtained by KNN.

**Table 8:** Sensitivity, Specificity, Precision, F1 score and accuracy in model test for high power set for clinical data only

Algorithm	Sensitivity (95% CI)	Specificity (95% CI)	Precision (95% CI)	F1 score	Accuracy (95% CI)
Fine tree	100.00 (95.73, 100.00)	91.49 (83.92, 96.25)	91.40 (84.56, 95.37)	95.51	95.53 (91.38, 98.05)
Medium tree	100.00 (95.73, 100.00)	91.49 (83.92, 96.25)	91.40 (84.56, 95.37)	95.51	95.53 (91.38, 98.05)
Discriminant	95.70 (89.35, 98.82)	95.35 (88.52, 98.72)	95.70 (89.52, 98.31)	95.70	95.53 (91.38, 98.05)
SVM	93.75 (84.76, 98.27)	71.30 (62.12, 79.35)	64.52 (57.51, 70.95)	76.44	79.33 (72.65, 85.01)
KNN	100.00 (95.94, 100.00)	95.56 (89.01, 98.78)	95.70, (89.51, 98.31)	97.80	97.77 (94.38, 99.39)
Neural Network (MLP)	94.12 (85.62, 98.37)	73.87 (64.68, 81.75)	68.82 (61.61, 75.21)	79.51	81.56 (75.10, 86.96)

*CI* Confidence interval

The results of Task IV presented in Table 9 showed the highest sensitivity, F1 score and overall accuracy was 100% obtained by Discriminant model, while the highest specificity and precision was 100% obtained by discriminant and Neural Network (MLP) models.

**Table 9:** Sensitivity, Specificity, Precision, F1 score and accuracy in model test for medium power

Algorithm	Sensitivity (95% CI)	Specificity (95% CI)	Precision (95% CI)	F1 score	Accuracy (95% CI)
Fine tree	98.81 (93.55, 99.97)	96.51 (90.14, 99.28)	98.81 (92.20, 99.83)	98.81	97.65 (94.09, 99.36)
Medium tree	98.81 (93.55, 99.97)	96.51 (90.14, 99.28)	96.51 (90.10, 98.83)	97.65	97.65 (94.09, 99.36)
Discriminant	100.00 (95.80,100.00)	100.00 (95.70,100.00)	100.00 (95.80, 100.00)	100.00	100.00 (97.85, 100.00)
SVM	97.70 (91.94, 99.72)	98.80 (93.47, 99.97)	98.84 (92.37, 99.83)	98.27	98.24 (94.93, 99.64)
KNN	98.78 (93.39, 99.97)	94.32 (87.24, 98.13)	94.19 (87.36, 97.43)	96.43	96.47 (92.48, 98.69)
Neural Network (MLP)	98.85 (93.76, 99.97)	100.00 (95.65, 100.00)	100.00 (95.80, 100.00)	99.42	99.41 (96.77, 99.99)

**Fig. 11:**
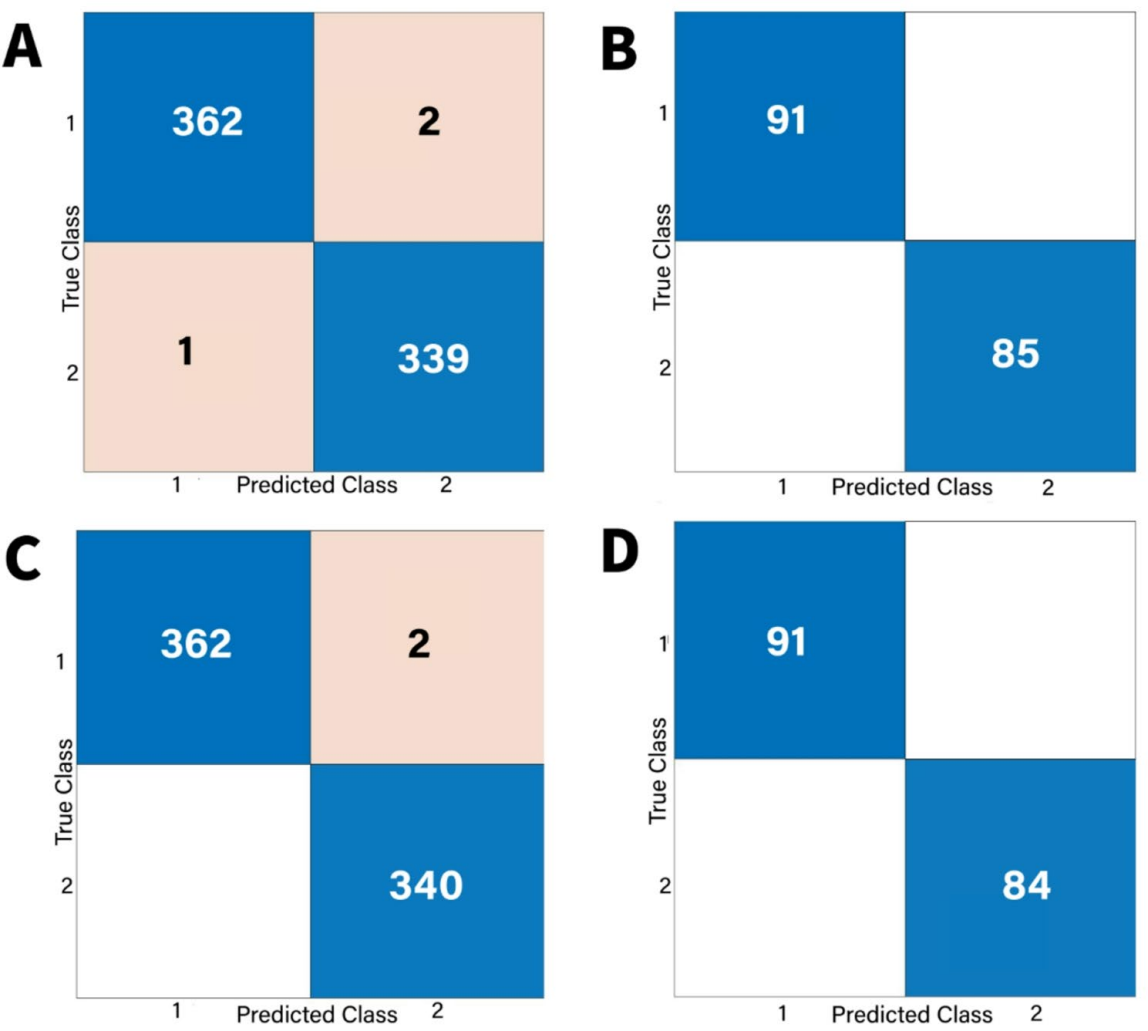
Confusion matrix of classification results for SVM model. **A** Classification matrix for model validation for task I. **B** Classification matrix for model testing for task (I) (**C**) Classification matrix for model validation for task (II) (**D**) Classification matrix for model testing for task II

**Fig. 12:**
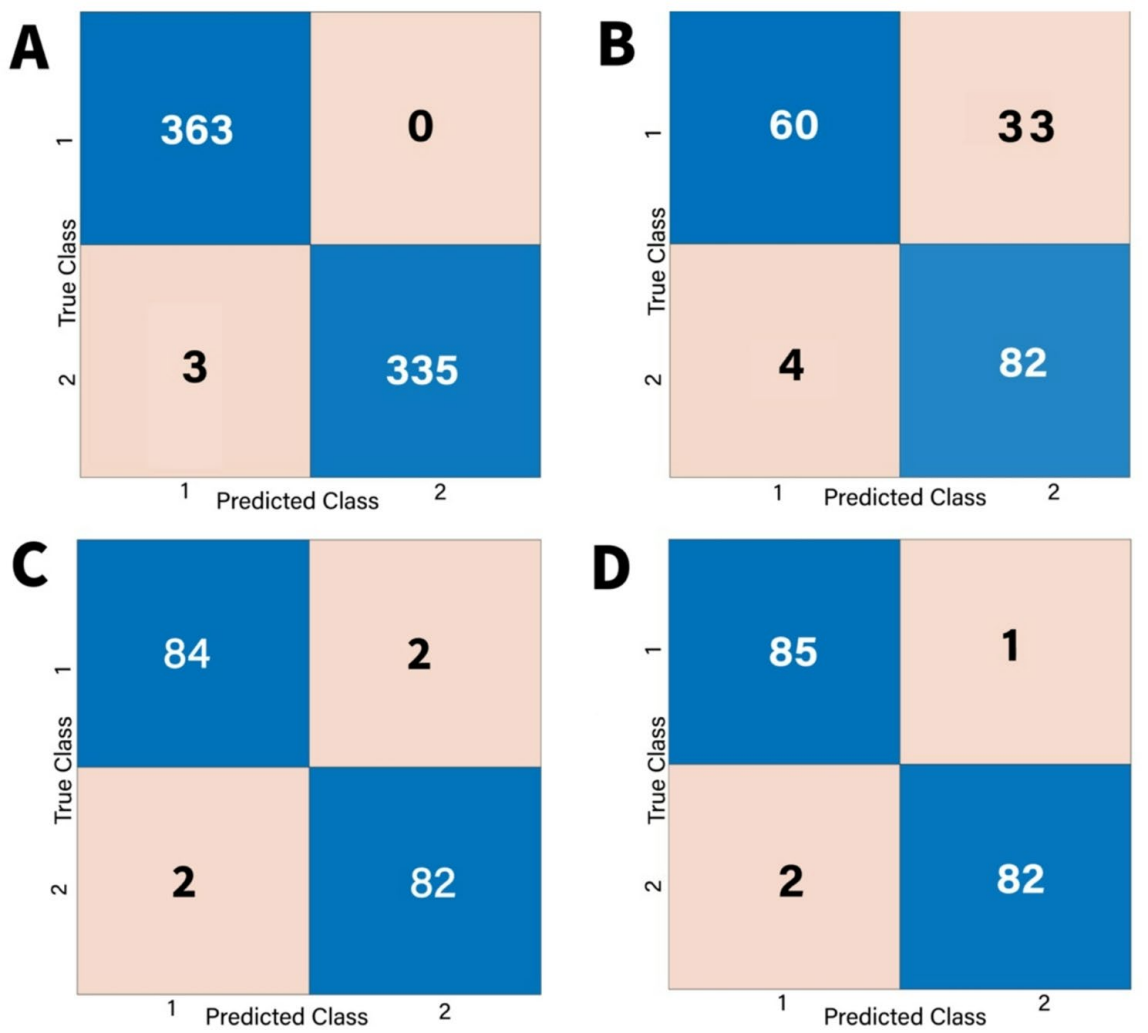
Confusion matrix of classification results for SVM model. **A** Classification matrix for model validation for task III. **B** Classification matrix for model testing for task III. **C** Classification matrix for model validation for task IV. **D** Classification matrix for model testing for task IV

The confusion matrices for all models were done for validation and testing. For SVM models, the results of confusion matrix were shown in Figs. 11 and 12. According to these results, sensitivity, specificity, precision and F1 score were calculated by the following formula:

1 $$\:\:\:\:\:\:\:\:\:\:\:\:\:\:\:\:\:\:\:\:\:\:\:\:Sensitivity=\frac{TP}{TP+\:FN}$$

2 $$\:Specificity=\frac{TN}{TN+\:FP}$$

3 $$\:Precision=\frac{TP}{TP+FP}$$

4 $$\:\:\:\:\:\:\:\:\:\:\:\:\:\:\:\:\:\:\:\:\:\:\:\:\:\:\:\:\:\:\:\:\:\:\:\:\:\:\:\:\:\:\:\:\:\:\:\:\:\:\:F1-Score=2x\frac{Precision\:x\:Recall}{Precision+Recall}$$

For both normal and OSCC histological images, TP (True Positive) and TN (True Negative) denote correctly classified images. Conversely, FN (False Negative) and FP (False Positive) indicate misclassified images within these categories [45] Figs. 11 and 12.

To demonstrate the added value of texture analysis, we performed McNemar test for accuracy among 4 tasks and report 95% confidence intervals. This step is crucial to substantiate the clinical utility of GLCM features beyond readily available clinical information which presented in Table 10.

**Table 10:** Difference between AI models and the oral pathologist’s diagnosis in detecting OSSC

Algorithm		Oral pathologist’s diagnosis		95% CI	*p* value
		Positive *n* (%)	Negative *n* (%)		
Task I					
Fine tree	**Positive**	91 (100.00)	0 (0.00.00)	(0.00, 0.00)	-
	**Negative**	0 (0.00)	84 (100.00)		
Medium tree	**Positive**	91 (100.00)	0 (0.00.00)	(0.00, 0.00)	-
	**Negative**	0 (0.00)	84 (100.00)		
Discriminant	**Positive**	91 (100.00)	0 (0.00.00)	(0.00, 0.00)	-
	**Negative**	0 (0.00)	84 (100.00)		
SVM	**Positive**	91 (100.00)	0 (0.00.00)	(0.00, 0.00)	-
	**Negative**	0 (0.00)	84 (100.00)		
KNN	**Positive**	91 (100.00)	0 (0.00.00)	(0.00, 0.00)	-
	**Negative**	0 (0.00)	84 (100.00)		
Neural Network (MLP)	**Positive**	91 (100.00)	0 (0.00.00)	(0.00, 0.00)	-
	**Negative**	0 (0.00)	84 (100.00)		
Task II					
Fine tree	**Positive**	91 (100.00)	0 (0.00.00)	(0.00, 0.00)	-
	**Negative**	0 (0.00)	84 (100.00)		
Medium tree	**Positive**	91 (100.00)	0 (0.00.00)	(0.00, 0.00)	-
	**Negative**	0 (0.00)	84 (100.00)		
Discriminant	**Positive**	91 (100.00)	0 (0.00.00)	(0.00, 0.00)	-
	**Negative**	0 (0.00)	84 (100.00)		
SVM	**Positive**	91 (100.00)	0 (0.00.00)	(0.00, 0.00)	-
	**Negative**	0 (0.00)	84 (100.00)		
KNN	**Positive**	91 (100.00)	0 (0.00.00)	(0.00, 0.00)	-
	**Negative**	0 (0.00)	84 (100.00)		
Neural Network (MLP)	**Positive**	91 (100.00)	0 (0.00.00)	(0.00, 0.00)	-
	**Negative**	0 (0.00)	84 (100.00)		
Task III					
Fine tree	**Positive**	85 (100.00)	8 (8.51)	(−7.50, −1.44)	0.01*
	**Negative**	0 (0.00)	86 (91.49)		
Medium tree	**Positive**	85 (100.00)	8 (8.51)	(−7.50, −1.44)	0.01*
	**Negative**	0 (0.00)	86 (91.49)		
Discriminant	**Positive**	89 (95.70)	4 (4.65)	(−3.10, 3.10)	1.00
	**Negative**	4 (4.30)	82 (95.35)		
SVM	**Positive**	60 (93.75)	33 (28.70)	(−22.42, −9.98)	< 0.001*
	**Negative**	4 (6.25)	82 (71.30)		
KNN	**Positive**	89 (100.00)	4 (4.44)	(−4.40, −0.07)	0.12
	**Negative**	0 (0.00)	86 (95.56)		
Neural Network (MLP)	**Positive**	64 (94.12)	29 (26.13)	(−19.91, −8.02)	< 0.001*
	**Negative**	4 (5.88)	82 (73.87)		
Task IV					
Fine tree	**Positive**	83 (98.81)	1 (1.19)	(−1.65, 1.65)	1.00
	**Negative**	1 (1.19)	83 (98.81)		
Medium tree	**Positive**	83 (98.81)	3 (3.49)	(−3.48, 1.12)	0.62
	**Negative**	1 (1.19)	83 (96.51)		
Discriminant	**Positive**	86 (100.00)	0 (0.00)	(0.00, 0.00)	-
	**Negative**	0 (0.00)	84 (100.00)		
SVM	**Positive**	85 (97.70)	1 (1.20)	(−1.41, 2.58)	1.00
	**Negative**	2 (2.30)	82 (98.80)		
KNN	**Positive**	81 (98.78)	5 (5.68)	(−5.15, 0.45)	0.22
	**Negative**	1 (1.22)	83 (94.32)		
Neural Network (MLP)	**Positive**	86 (98.85)	0 (0.00)	(−0.56, 1.74)	1.00
	**Negative**	1 (1.15)	83 (100.00)		

McNemar test

*CI* Confidence interval

The crucial comparison is between the model using Clinical Data ONLY (Task III) and the model using Clinical + Image/GLCM Data (Task I). This directly shows the added benefit of texture analysis. For all six tested machine learning algorithms, the combined model (Task I) was statistically superior to the Clinical-Only model (Task III). The models that included GLCM features (Task I) achieved 100% accuracy, while the Clinical-Only models (Task III) had significantly lower accuracy, with gains ranging up to over 20% points (e.g., for the SVM model). The 95% Confidence Intervals (CIs) for the high-performing combined models (Task I) do not overlap with the CIs for the lower-performing Clinical-Only models (Task III). This is the formal statistical proof that the Image (GLCM) features provide significant, non-redundant value that goes beyond the clinical baseline, thereby substantiating their clinical utility.

We also noted the performance of the Image/GLCM Data ONLY (Task II) models. The Image-Only models (Task II) achieved a perfect 100% accuracy across all algorithms, matching the perfect performance of the combined Clinical + Image models (Task I). This demonstrates that the GLCM features are highly powerful and robust, as they were sufficient on their own to achieve maximum performance. The addition of clinical data (Task I) does not statistically degrade the perfect accuracy achieved by the texture features.

The results of Task I, II and IV are highly consistent in the image-based split and patient-based split. In patient-based split, the performance of the models across the three datasets was exceptionally high, with the maximum Test Accuracy reaching 100.00% in two of the evaluated scenarios. Specifically, the models corresponding to Task I and Task II, indicating flawless classification on their respective test sets. For Task IV, demonstrated a slightly lower yet highly robust maximum Test Accuracy of 98.27%. Final metrics at the patient level were reported as Additional file 1 for Task I, Additional file 2 for Task II and Additional file 3 for Task IV.

#### Task III (Clinical data Only)

 The accuracy of the clinical-only model decreased significantly after the patient-wise split which presented in Table 4 for validation and Table 8 for test.

Furthermore, to demonstrate the consistency and robustness of our model within the cohort, the final reported results are supported by four additional, independent full cross-validation runs, each utilizing a new and distinct patient-level test set. The highly consistent performance across all five runs validates the stability of the final models. The detailed results of these four additional validation runs are provided in Additional Files 4, 5, 6, and 7.

### Clinical feature importance and ablation results

To mitigate the risk of patient-specific bias and confounding, we implemented two key procedural changes:

3. Patient-Level Data Splitting: All cross-validation and final test sets were strictly separated at the patient level. This ensured that images from the same patient were never split across both the training and testing partitions. Table 8.
4. Clinical-Only Model Robustness Check: To validate the discriminatory power of clinical features independent of image count per patient, we performed an additional test on the Clinical-Only model using a highly constrained dataset consisting of only one randomly selected image per patient. The maintenance of good performance in this restrictive setting is detailed in Additional File 8.

**Fig. 13:**
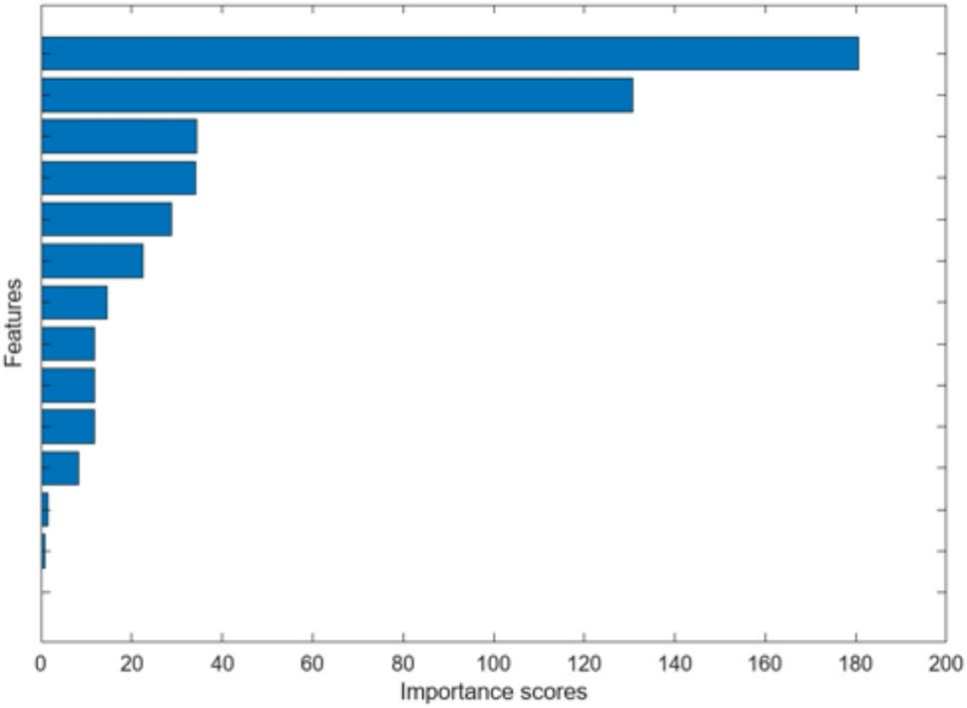
Clinical feature importance scores sorted using Chi2 algorithm domenstrating the most two important features which are age followed by anatomical site of anterior giniva

The feature importance analysis, conducted using the Chi-Square (χ2) algorithm, confirmed Age and Anterior Gingiva as the two most influential clinical features Fig. 13.

A comprehensive quantification of all clinical feature importance scores is provided in Additional File 9.

Based on this ranking, which identified Age and Anterior Gingiva as the top two influential features, we performed three distinct ablation tests:

5. Ablation of Top 1 Feature (Age): Removal of Age alone (Additional File 10).
6. Ablation of Top 2 Feature (Anterior Gingiva): Removal of Anterior Gingiva alone (Additional File 11).
7. Ablation of Top 1 and Top 2 Features (Age & Anterior Gingiva): Simultaneous removal of both features (Additional File 12).

The results of the ablation experiments, summarizing the change in maximum Test Accuracy for our five primary model types, are presented in Table 11.

**Table 11:** Shows the maximum test accuracy achieved by each of those model types in the “All Features” scenario, and how their best performance changed after the ablation of first, second and then two features

Model Type	All Features Accuracy (%)	Top 1 Removed (Age) Accuracy (%)	Top 2 Removed (Anterior Gingiva) Accuracy (%)	1Top 2 Removed (Age & Anterior Gingiva) Accuracy (%)
KNN	97.77	84.92	81.56	65.92
Discriminant	95.53	84.36	81.56	88.27
Tree	95.53	87.15	79.33	88.27
Neural Network (MLP)	81.56	65.92	79.33	88.27
SVM	79.33	88.27	79.33	88.27

The ablation analysis demonstrates the critical role of both features in achieving peak performance. The removal of Anterior Gingiva alone resulted in the most significant single-feature decline across high-performing models, specifically reducing the top KNN model’s accuracy from 97.77% to 81.56% (a 16.21% drop). Similarly, the removal of Age caused substantial degradation, particularly for the Neural Network (MLP), which dropped by 15.64%. Crucially, the combined removal of both features resulted in the most severe performance loss overall, confirming their synergistic contribution and emphasizing their necessity for maximum classification accuracy.

## Discussion

Oral Squamous Cell Carcinoma (OSCC) represents approximately 90% of all oral neoplasms, yet its mortality rate has remained stubbornly high since the 1980 s [3–5]. While histopathological examination remains the gold standard for OSCC diagnosis and grading [8, 9], this manual process is often subjective, time-consuming, and prone to pathologist fatigue and inter-observer variability [10–12]. These limitations highlight a critical need for more precise, rapid, and standardized diagnostic methods to improve patient outcomes [6].

To address these challenges, we proposed the integration of AI to enhance the accuracy and efficiency of OSCC diagnosis [13]. Our study explored both traditional machine learning and deep learning approaches, leveraging AI’s ability to analyze medical images more objectively [27]. Specifically, we focused on texture-based analysis of histopathological slides using the Gray Level Co-occurrence Matrix (GLCM) algorithm [31]. This technique allowed us to extract distinct texture features that correlate with the characteristics pathologists traditionally use, aiming to provide a robust comparison of AI model precision and accuracy in assisting oral pathologists.

The results presented in our work revealed significant differences in texture- based image features, including Autocorrelation, Contrast, Difference variance, Cluster Shade, Dissimilarity, Energy, Entropy, Sum entropy, Difference entropy, Homogeneity, Maximum Probability, and Information measure of correlation, between the normal and OSCC groups. Furthermore, the study highlighted the high performance of machine learning models in classifying these groups.

### Texture analysis and image features

The autocorrelation results indicate that OSCC images exhibit a finer texture (smaller range: *<*40) compared to normal tissue (*<* 62). This suggests that cancerous regions may have more irregular structural patterns, leading to reduced autocorrelation [40]. Similarly, higher contrast and difference variance values in the OSCC group compared to the normal group reflect greater local intensity variations, which may correspond to tumor heterogeneity [40, 41]. Entropy measures further support this observation, with OSCC samples showing higher entropy compared to normal tissue indicating increased randomness and complexity in cancerous regions [40, 41]. Dissimilarity was also elevated in OSCC compared to normal tissue, suggesting greater intensity disparities, which may be linked to abnormal cellular arrangements [40].

Energy and maximum probability were lower in OSCC compared to normal tissue, implying reduced uniformity in pixel intensity co-occurrence [32]. Homogeneity was also lower in OSCC compared to normal tissue, reinforcing the idea of disrupted tissue architecture in malignancies [42]. Cluster shade, a measure of texture asymmetry, showed a wider range in OSCC compared to normal tissue, suggesting greater irregularity in tumor texture patterns [44].

In comparing the texture feature extraction results between our study focusing on OSCC and Cheung et al. [32] work focusing on glioblastoma (GBM), several similarities and dissimilarities emerge across various image features. For autocorrelation, our work’s normal group (for the first and second set together) exhibited ranges of 27 to 62, with the OSCC group showing ranges of 20 to 40, generally suggesting finer details with smaller values. The GBM study’s normal group showed a narrower range of 15 to 31, and the GBM group 10.5 to 57, with the common understanding that smaller values indicate finer details.

Regarding local intensity variation, our work’s normal group (for the first and second set together) had ranges of 0.06 to 0.6, while the OSCC group showed 0.13 to 0.7, where high contrast reflects rapid intensity changes. Cheung et al. [32] highlighted contrast, with normal groups typically below 0.4 and GBM groups from 0.5 to 3.5, indicating higher contrast in GBM.

For entropy features, our study noted that higher entropy signifies greater complexity; the normal group’s entropy, sum entropy, and difference entropy (for the first and second set together) typically ranged from 0.6 to 2.8, 0.6 to 2.35, and 0.22 to 0.85, while the OSCC group generally showed higher ranges (e.g., entropy 1.3 to 2.8 for the first set). Cheung et al. [32] echoed this, with normal groups typically below 1.52 (entropy), 1.3 (sum entropy), and 0.6 (difference entropy), and GBM groups generally showing higher values above 1.6, consistent with the notion that higher entropy indicates greater complexity.

In terms of dissimilarity, our normal group (for the first and second set together) ranged from 0.06 to 0.45, with the OSCC group from 0.13 to 0.53, implying that greater dissimilarity corresponds to more distinct average brightness levels. Cheung et al. [32] also observed that normal groups were below 0.3 and GBM groups higher than 0.3, aligning with the concept that higher dissimilarity means a greater difference in average image values.

For energy, our study’s normal group (for the first and second set together) ranges were 0.08 to 0.58, while the OSCC group showed 0.08 to 0.32, with higher energy indicating a greater tendency for neighboring intensities to co-occur frequently. Cheung et al. [32] similarly found normal groups higher than 0.28 and GBM groups lower than 0.28. Maximum probability in our work for the normal group (for the first and second set together) was 0.12 to 0.88 and for OSCC were from 0.11 to 0.6. Cheung et al. [32] normal groups were higher than 0.5, and GBM groups lower than 0.43.

Homogeneity in our work showed that normal group (for the first and second set together) ranged from 0.868 to 0.965, and OSCC group ranged from 0.77 to 0.93 with increasing values indicating more uniform intensity. Cheung et al. [32] similarly identified normal groups with homogeneity 1 higher than 0.85 and GBM groups lower than 0.85, confirming that higher values suggest greater uniformity.

Lastly, cluster shade in our normal group (for the first and second set together) ranged from − 29 to 20, with the OSCC group showing wider ranges of −39 to 35. Overall, both papers utilized similar texture features, and while the exact numerical ranges differ due to the distinct diseases (OSCC vs. GBM) and possibly different image datasets or processing, the interpretative trends for how these features characterize normal versus diseased tissues often align (e.g., higher entropy for more complex disease patterns, lower homogeneity for less uniform disease textures) [32].

Machine Learning Model Performance This study evaluated multiple machine learning (ML) models and a deep learning approach “Neural Network (MLP)” for classifying normal vs. OSCC tissue based on texture features.

The classification results for (Task I–IV) demonstrated exceptional performance, with several models achieving 100% sensitivity, specificity, precision, F1 score, and overall accuracy in both validation and testing phases. Notably:

kNN and Discriminant Analysis emerged as the most robust for Tasks III–IV, possibly due to: - kNN’s non-parametric nature (effective for small datasets). - Discriminant’s statistical stability with low-dimensional features. We recorded excellent results for the high-power set with clinical data (Task I), high power set without clinical data (Task I), Clinical data only (Task III) with almost no difference between all models in all tasks.

For Task II, images examined at 400x magnification, our KNN and SVM models, based on GLCM texture feature extraction, achieved perfect accuracy (100.00%) in OSCC. This significantly outperforms the 84.2% and 89.7% accuracies previously reported by Elazab et al. [46] in brain tumor, highlighting the robustness of our approach under these specific conditions examining histopathological images.

For Task IV, images examined at 100x magnification, our SVM model achieved excellent classification performance with an accuracy of 98.24%, F1 score of 98.27%, precision of 98.84%, specificity of 98.8%, and sensitivity of 97.7%. While Cheung et al. [32] demonstrated high specificity (99.73%) with their SVM model, our approach showed superior performance in accuracy (98.24%), F1 score (98.27%), precision (98.84%), and sensitivity (97.7%), indicating a more balanced and effective diagnostic capability. Our Neural Network (MLP) model demonstrated exceptionally high diagnostic performance, particularly at higher magnifications. At 400x magnification, the model achieved perfect scores across all evaluated metrics: 100% for sensitivity, specificity, precision, F1 score, and accuracy. At 100x magnification, performance remained robust, yielding 98.85% sensitivity, 100% specificity, 100% precision, 99.42% F1 score, and 99.4% accuracy. These results notably surpass those reported by Soni et al. [47] who using an improved EfficientNetB0 architecture for OSCC detection, demonstrated 92.2% sensitivity, 91% specificity, 91.1% precision, 92.3% F1 score, and 91.1% accuracy. The marked difference in performance underscores the enhanced diagnostic capability of our Neural Network (MLP) model, suggesting its potential for more precise and reliable clinical application in histopathological analysis.

The evaluation of our Neural Network (MLP) model on 100x magnification images revealed high levels of accuracy, with both validation and test accuracies at 99.41%. This represented a significant advancement over previous work, such as that by Rahman et al. [48] which applied a customized AlexNet to histopathological images of OSCC uploaded from kaggle [49], reported a validation accuracy of 97.6% and a testing accuracy of 90.06%. Unlike studies relying on publicly available datasets, our approach gained robustness from utilizing our own unique sets of histological images which were obtained from the Egyptian population.

Our Neural Network (MLP) model achieved remarkable performance in differentiating between OSCC and normal tissue using 400x magnification images, demonstrating 100% precision and sensitivity. This significantly outperforms existing models, as exemplified by the PiT model (de Lima et al.) [39], which reported 93% precision and 92% sensitivity for a similar task. Furthermore, when integrating clinical data, our network maintained perfect precision and sensitivity (100%) in discriminating between normal and OSCC cases. This strong performance contrasts with the ViT model fusion with MetaBlock, which achieved 91.8% precision and 91.2% sensitivity in differentiating between normal tissue and tissue with dysplasia.

We observed that traditional ML models (e.g., kNN, SVM) performed equally well or better than the Neural Network (MLP) in most tasks. This suggests that handcrafted texture features provide sufficient discriminative power, reducing the need for complex deep learning in this specific context. The consistency between validation and testing phases underscores the reliability of these models. However, there are limitations to this study; we acknowledge that the data originates from a single center, which may limit generalizability. While we addressed the risk of internal overfitting by implementing a strict patient-wise split and confirming the robustness of our results across multiple independent patient test sets (as detailed in the Methods section and Additional Files 9–12), the ultimate confirmation of model generalizability will require validation on an external or multi-center test set, which we have identified as a priority for future work. Additionally, integrating these texture features with other diagnostic markers (e.g., genomic or proteomic data) could enhance predictive power.

### Recommendation

 In resource-constrained settings (e.g., low-power devices), prioritize KNN or SVM. For scalable systems, consider hybrid approaches (e.g., Neural Network (MLP) model + texture features). This comparison underscores that model selection should depend on data type, computational resources, and clinical requirements rather than defaulting to deep learning.

Future work could explore deep learning-based texture analysis for finer feature extraction where the deep learning models can learn hierarchical features automatically if raw image data (not just extracted features) were used. As well as they are better suited for larger, more diverse datasets.

## Conclusions

This study successfully identified distinct textural differences between normal and OSCC tissues using statistical image features. Machine learning models achieved outstanding classification performance; The strong performance of these models indicates potential for computer-aided diagnosis (CAD) systems in early OSCC detection. The ability to distinguish normal from malignant tissue with near-perfect accuracy could reduce reliance on subjective visual assessments and improve diagnostic efficiency.

## Supplementary Information

Additional file 1. Table of results for High power set with clinical data with 5-fold cross validation with patient split.

Additional file 2. Table of results for High power set without clinical data with 5-fold cross validation with patient split.

Additional file 3. Table of results for Medium power set with 5-fold cross validation with patient split.

Additional file 4. Table of results for High power set with 5-fold cross validation with patient split with a new, separate test set of patients.

Additional file 5. Table of results for High power set with 5-fold cross validation with patient split with a new, separate test set of patients.

Additional file 6. Table of results for High power set with 5-fold cross validation with patient split with a new, separate test set of patients.

Additional file 7. Table of results for High power set with 5-fold cross validation with patient split with a new, separate test set of patients.

Additional file 8. Tables of results for clinical data only with cross validation with only one image with only one clinical data for every patient.

Additional file 9. A more detailed analysis of the importance of all clinical features, determined using a Chi-Square test.

Additional file 10. Table of results for clinical data only with cross validation with patient split after ablation of the age feature.

Additional file 11. Table of results for clinical data only with cross validation with patient split after ablation of the Anterior gingiva feature.

Additional file 12. Table of results for clinical data only with cross validation with patient split after ablation of the Age and Anterior gingiva feature.

## Data Availability

The datasets used and analyzed during the current study are available from the corresponding author on reasonable request.

## Abbreviations

Ai: Artificial intelligence
FT: Fine Tree
GBM: Glioblastoma
GLCM: Gray Level Co-occurrence Matrix
KNN: K-Nearest Neighbors
LD: Linear Discriminant
MLP: Multilayer perceptron
MT: Medium Tree
OSCC: Oral squamous cell carcinoma
SVM: Support Vector Machine
WHO: World Health Organization
