# Supplementary material for: Texture-based image feature analysis for the classification of oral squamous cell carcinoma using machine learning approach
Source: BMC Oral Health. 2025 Dec 27;26:105. doi: 10.1186/s12903-025-07482-1 (PMC12809959; doi:10.1186/s12903-025-07482-1)
Supplement: Supplementary file 9 — Additional file 9. A more detailed analysis of the importance of all clinical features, determined using a Chi-Square test. [file 12903_2025_7482_MOESM9_ESM.docx]

A more detailed analysis of the importance of all clinical features, determined using a **Chi-Square test**


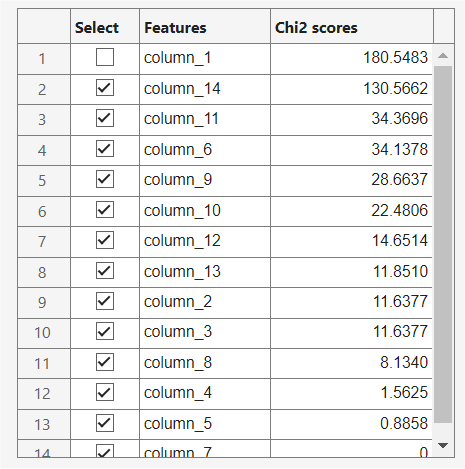


Column_1 Age

Column_2 Male

Column_3 Female

Column_4 Buccal mucosa/Posterior buccal mucosa

Column_5 Mandibular alveolar ridge

Cloumn_6 Lateral surface of tongue

Column_7 Ventral surface of tongue

Column_8 Upper lip

Column_9 Left maxilla/Anterior maxilla/Maxillary alveolar ridge

Column_10 Hard palate/Palatal surface of left maxilla

Column_11 Retromolar area

Column_12 Zygoma/Condyle

Column_13 Dorsal surface of tongue

Column_14 Anterior gingiva
